# miR-30c-5p Alleviated Pyroptosis During Sepsis-Induced Acute Kidney Injury *via* Targeting TXNIP

**DOI:** 10.1007/s10753-020-01323-9

**Published:** 2020-09-05

**Authors:** Xiang Li, Linya Yao, Xueming Zeng, Bing Hu, Xi Zhang, Jun Wang, Runyu Zhu, Qiwei Yu

**Affiliations:** Department of Urinary Surgery, Kunshan Hospital of Traditional Chinese Medicine, Kunshan, 215300 Jiangsu China

**Keywords:** sepsis-induced acute kidney injury, pyroptosis, miR-30c-5p, TXNIP

## Abstract

Sepsis-induced acute kidney injury (SAKI) is a common complication of hospitalized patients, often leading to unacceptable mortality. Limited effective treatment or diagnosis biomarkers are available and the underlying mechanism remains unclear. The miR-30c-5p is considered as a critical mediator of kidney diseases and aberrantly decreased in patients with SAKI, while the mechanism is still unclear. For this purpose, the role of miR-30c-5p in SAKI has been investigated in this study. Here, we first confirmed that miR-30c-5p expression decreased in our septic models and was associated with the activation of NLRP3/caspase-1-mediated pyroptosis. Overexpression of miR-30c-5p alleviated the kidney injury *via* suppressing HK-2 cell pyroptosis. Furthermore, we identified that TXNIP was a direct target of miR-30c-5p. Upregulation of miR-30c-5p repressed the expression of TXNIP, which inhibited NLRP3, ASC, and caspase-1 expression, as well as secretion of inflammatory cytokines. In conclusion, our data suggested that miR-30c-5p negatively controlled the NLRP3 signal pathway-related pyroptosis and sepsis-induced injury *via* TXNIP, indicating that this axis might be a positive therapeutic target for the patient with SAKI.

## INTRODUCTION

Sepsis is a complex syndrome characterized by the dysregulated inflammatory response to the infection in the host [1, 2]. Sepsis-induced acute kidney injury (SAKI), a severe complication during sepsis formation, is observed in most hospitalized patients, especially in the elderly [3, 4]. It is accompanied by the function and organ disorder in the kidney and is responsible for the high mortality in septic patients [5]. Unfortunately, no effective treatment or diagnosis approaches are available for SAKI [6]. Thus, elucidating the underlying molecular basis and developing the novel therapeutic option for SAKI prevention remains essential.

MicroRNA (miRNA) is a kind of non-coding RNAs with about 22–26 nucleotides in length that involves in several pathophysiological progress and diseases *via* binding to the 3’-UTR of one or multiple downstream genes to regulate the target gene expression at the post-transcriptional level [7–9]. Recent studies have implicated microRNAs as an essential factor controlling sepsis-induced injury formation [10, 11]. One previous study found that overexpression of miR-21 could protect the kidney cell from sepsis-induced apoptosis, thus alleviating the sepsis-induced acute kidney injury [12]. Another study also determined that miR-204 reduce the IL-6R expression to alleviate LPS-induced mesangial cell apoptosis and oxidative stress accumulation in SAKI [13]. Of interest, miR-30c-5p, which has been proved as an essential mediator of kidney injury, was able to inhibit the expression of tumor necrosis factor-alpha (TNF-α) and multiple inflammatory cytokines in rats [14, 15]. Overexpression of miR-30c-5p could also significantly increase human renal tubular epithelial cells (HK-2 cells) apoptosis to inhibit kidney stones formation [16]. Intriguingly, the study of Pan *et al.* observed that miR-30c-5p was dramatically decreased in patients with SAKI as compared with the healthy people [17]. However, the mechanism by which miR-30c-5p regulates the HK-2 cell death in SAKI remains elusive.

For this purpose, in the present study, we first established a SAKI mice model by LPS injection and stimulated the septic model *in vitro* using LPS-treated HK-2 cells. Subsequently, the expression pattern of candidate miRNA, miR-30c-5p, was identified using qRT-PCR. Next, the molecular basis for the role of miR-30c-5p in SAKI was determined using our *in vivo* and *in vitro* models. We found that miR-30c-5p was significantly downregulated in HK-2 cells upon LPS stimulation, which further facilitated HK-2 cell pyroptosis and aggravated kidney injury *via* negatively regulating thioredoxin-interacting protein (TXNIP) expression. Our findings contribute to the development of novel therapeutic approaches for SAKI.

## METHODS

### Animal Model

All the animal experiments were approved by the ethics committee of XXX. C57BL/6 mice (8–12 weeks old) were obtained from the Animal Center of Nanjing University and housed in a temperature and humidity-controlled condition with free access to food and water. The septic model was induced by injecting intraperitoneally of 16 mg/kg LPS (*Escherichia coli* O111:B4, Sigma, USA) as the previous study [18]. The sham group was received an equal volume of PBS (500 μL). Twenty-four hours after injection, the mice were killed by CO_2_ inhalation, with the kidney tissues isolated and serum drawn.

### Cell Culture and Treatment

Human renal tubular epithelial cell line HK-2 were obtained from ATCC and cultured in DMEM medium (Gibco, USA) with 10% fetal bovine serum, 100 U/mL penicillin and streptomycin (Thermo Fisher, USA), as well as 0.05 mg/mL bovine pituitary extract (BPE) and 5 ng/mL epidermal growth factor (EGF, Gibco, USA). To establish the septic model *in vitro*, HK-2 cells were incubated with 1 μg/mL LPS for 4 h. After the replenishment of fresh medium, 3 mM ATP (Sigma, USA) was added to cells and incubated for 1 h.

### Hematoxylin and Eosin Staining

The renal tissues were fixed in 10% formalin for 24 h and then embedded into the paraffin, followed by cutting into sections with 5 μm. According to the manufacturer’s protocol, the sections were stained by hematoxylin and eosin solution (Beyotime, China). The images were captured under the light microscope (Zeiss, Germany).

### Immunohistochemical Assay

Upon deparaffinization, the sections were subjected to citrate to retrieve antigen, followed by and blocked by 5% BSA buffer. Afterward, the sections were treated with primary antibody against GSDMD-N (1:300, Abcam, USA) or TXNIP (1:300, Abcam, USA) at 4 °C overnight. The sections were then incubated with biotinylated secondary antibody (30–40 μL, Abcam, USA) for 30 min at room temperature. The signals were developed using 3,3′-diaminobenzidine tetrahydrochloride peroxidase substrate kit (Vector Laboratories) following the manufacturer’s instructions. Nuclei were stained by hematoxylin. Finally, the positive signals were observed under a light microscopy (Olympus, Japan).

### ELISA

The content of IL-18, IL-6, TNF-α, and IL-1β in the serum and cell supernatant was detected by the commercial kits (Jiancheng, China). The absorbance of each sample was recorded at 450 nm using a microplate reader (Bio-Rad, USA).

### Western Blotting

Total proteins from kidney tissues or cells were extracted using RIPA buffer (Thermo Fisher, USA), and the concentration was measured by the BCA kit (Thermo Fisher, USA). Equal protein (30 μg/lane) were loaded and separated by the SDS-PAGE, followed by transfected onto the polyvinylidene difluoride (PVDF) membrane. After blocked by 5% bovine serum albumin (BSA) solution for 2 h at room temperature, the membranes were incubated with the primary antibodies against GSDMD (1:1000, Abcam, USA), GSDMD-N (1:1000, Abcam, USA), NLRP3 (1:1000, Abcam, USA), caspase-1 (1:1000, Abcam, USA), ASC (1:2000, Abcam, USA), IL-1β (1:2000, Abcam, USA), IL-18 (1:1000, Abcam, USA), TXNIP (1:1000, Abcam, USA), and GAPDH (1:5000, Abcam, USA) at 4 °C overnight, followed by treating the related secondary antibodies (1:5000, Abcam, USA) for 2 h at room temperature. The membranes were detected using the ECL kit (Thermo Fisher, USA) and analyzed by the ImageJ software.

### Transfection

The miR-30c-5p mimic, inhibitor, and related controls; the TXNIP knockdown; overexpression vectors (TXNIP-KD, TXNIP-OE); and empty vector were purchased from Synthgene (Nanjing, China). For the *in vitro* transfection, HK-2 cells were plated onto the 6-well plate at a density of 10^5^ cells per well. The molecules (50 nM of all) were mixed with Lipofectamine 2000 (Invitrogen, USA) and incubated with HK-2 cells for 48 h following the manufacturer’s instructions. For the *in vivo* transfection, 5 μg reagents were mixed with 8 μL Entranster™ reagent (Engreen, Beijing, China) and injected into the renal parenchyma of the mice at five random sites. Forty-eight hours after injection, the mice were used for further study.

### qRT-PCR

Total RNA from kidney tissues or cells was extracted using TRIzol reagent (Thermo Fisher, USA) and reverse transcribed into cDNA using the PrimeScript RT Master Mix Kit or miR^TM^ First-Strand Synthesis kit (TaKaRa, Japan). According to the manufacturer’s instructions, the expression of genes and miRNAs was analyzed on a qRT-PCR platform (Bio-Rad, USA) using SYBR Green (Bio-Rad, USA). The data was normalized by GAPDH or U6 and analyzed by the 2^−ΔΔCT^ method. The primer sequences were presented as follows: miR-30c-5p (human and mouse): forward 5′-GCGCGTGTAAACATCCTACACT-3′, reverse 5′-AGTGCAGGGTCCGAGGTATT-3′; NLRP3 (human): forward 5′-GATCTTCGCTGCGATCAACA-3′, reverse 5′-GGGATTCGAAACACGTGCATTA-3′; caspase-1(human): forward 5′- GCCTGTTCCTGTGATGTGGAG-3′, reverse 5′-TGCCCACAGACATTCATACAGTTTC-3′; ASC (human): forward 5′-CTACCTGGAGACCTACGGCG-3′, reverse 5′-TTTCCGGTAGAGCAGCTTTGT-3′; GAPDH(human): forward 5′-GCACCGTCAAGGCTGAGAAC-3′, reverse 5′-TGGTGAAGACGCCAGTGGA-3′; U6(mouse): forward 5′-GCTCGCTTCGGCAGCACAT-3′, reverse 5′-ATGGAACGCTTCACGAAT-3′; U6(human): forward 5′-GAAGCGCGGCCACGAG-3′, reverse 5′-AGTGCAGGGTCCGAGGTATT-3′.

### Immunofluorescence Assay

The cells were fixed by 4% paraformaldehyde solution for 10 min and blocked with 5% BSA solution at room temperature for 1 h. Next, the cells were treated with primary antibody against caspase-1 (1:1000, Abcam, USA) at 4 °C overnight. After incubation with related secondary antibody conjugated with Alexa Fluor 488 for 30 min at room temperature, the positive signals were observed under a fluorescence microscopy (Olympus, Japan).

### Luciferase Assay

The HEK 293T cells were purchased from ATCC and maintained in the DMEM medium (Gibco, USA). The wildtype (WT) and mutant (MUT) TXNIP 3’-UTR were synthesized into the pmirGLO Dual-Luciferase miRNA Target Expression Vector (Promega, USA). According to the manufacturer’s instructions, the luciferase report and miR-30c-5p mimic or mimic control (mimic-NC) were co-transfected into HEK 293T cells using Lipofectamine 3000. The luciferase activity was detected using the Dual-Luciferase® Reporter Assay System Protocol (Promega, USA).

### Statistical Analysis

All data were analyzed by GraphPad Prism 8.0 software and shown as the mean ± SD for three experiments independently. Statistical analysis was measured by a two-tailed Student’s *t* test. *p* < 0.05 was considered statistically significant.

## RESULTS

### Pyroptosis Was Induced During Sepsis-Induced AKI Formation

**Fig. 1:**
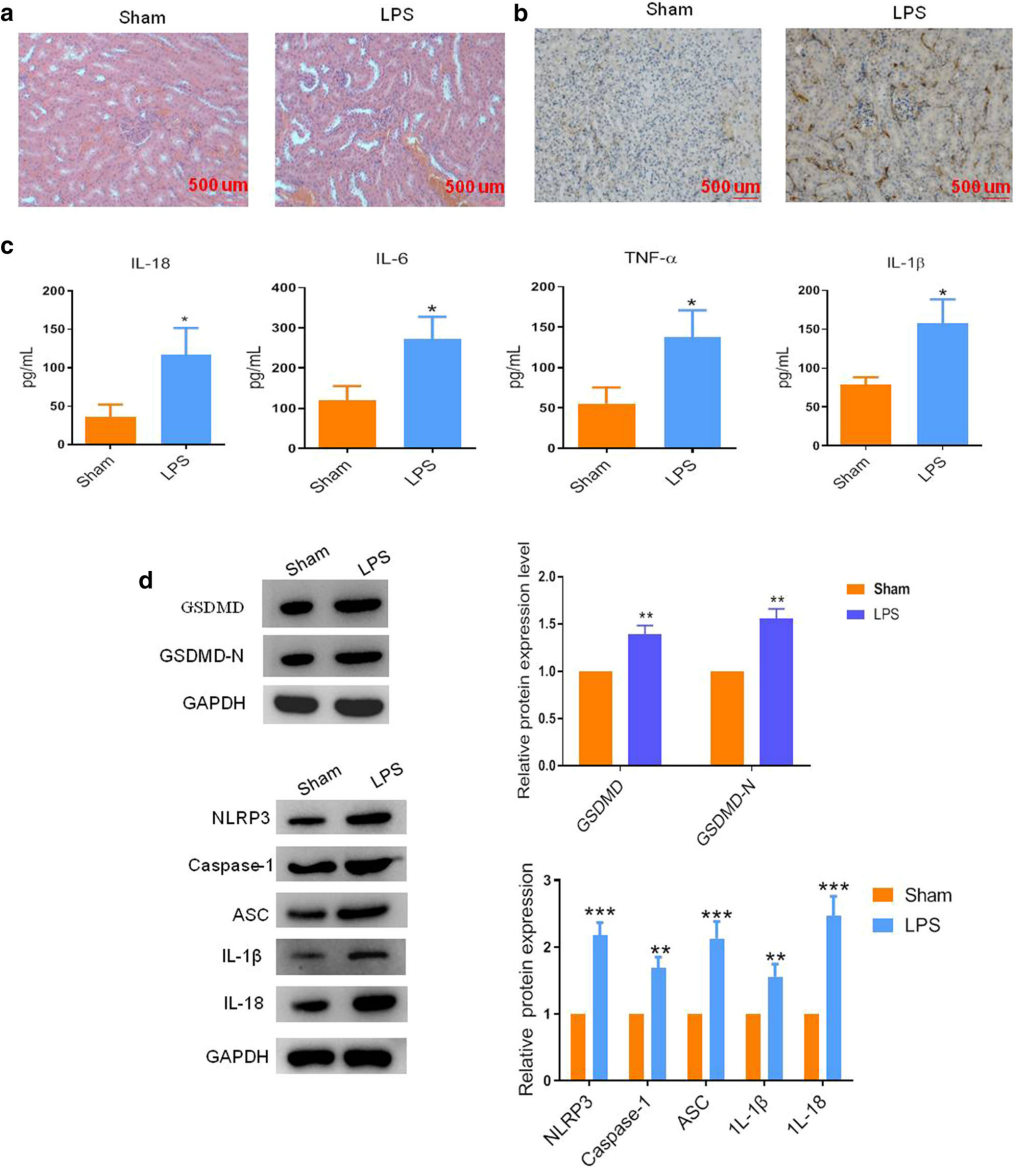
Pyroptosis was initiated in the kidney tissue of lipopolysaccharide (LPS)-derived septic mice. **a** Representative images of H&E staining and **b** IHC assay of GSDMD-N in the renal tissues after different treatments. Bar, 500 μm. **c** The concentration of serum IL-18, IL-6, IL-1β, and TNF-α in LPS-derived septic mice and the sham mice were determined by ELISA assay. **d** Representative blots and statistical analyses were shown for the expression of GSDMD, GSDMD-N, NLRP3, caspase-1, ASC, IL-1β, IL-18, and GAPDH in the mice with or without LPS treatment. The data represented the mean ± SD of three biological replicates, *p < 0.05, **p < 0.01, and ***p < 0.001 *vs.* the sham group.

Using the LPS-induced mouse model of SAKI, we first evaluated the state of renal epithelial cell death in SAKI. As shown in Fig. 1a, compared with the sham group, abundant tubular cavity expansion and massive vacuolar degeneration of tubular epithelial cells were presented in the septic mice. To identify whether pyroptosis was induced during SAKI, the expression of GSDMD-N, a marker of pyroptosis, was detected by IHC assay. As shown in Fig. 1b, LPS stimulation increased the GSDMD-N expression in the septic mice than the sham group. Besides, following the AKI, the inflammatory factors, including TNF-α, IL-18, IL-6, and IL-1β, were both significantly elevated in the serum of septic mice (Fig. 1c). Then we further checked the protein markers of pyroptosis in the renal tissues. The western blot analysis showed that GSDMD was activated in the LPS-exposed mice, as evidence by a 1.6-fold increase in the expression of GSDMD-N, compared with the sham group. Consistently, the protein expression levels of pyroptosis-related genes, including NLRP3, caspase-1, ASC, IL-1β, and IL-18, were markedly elevated after injury (Fig. 1d). These data illustrated that LPS treatment could trigger renal epithelial cell pyroptosis during sepsis-induced AKI formation.

### miR-30c-5p Was Downregulated in the Kidney Tissues of Septic Mice and HK-2 Cells

**Fig. 2:**
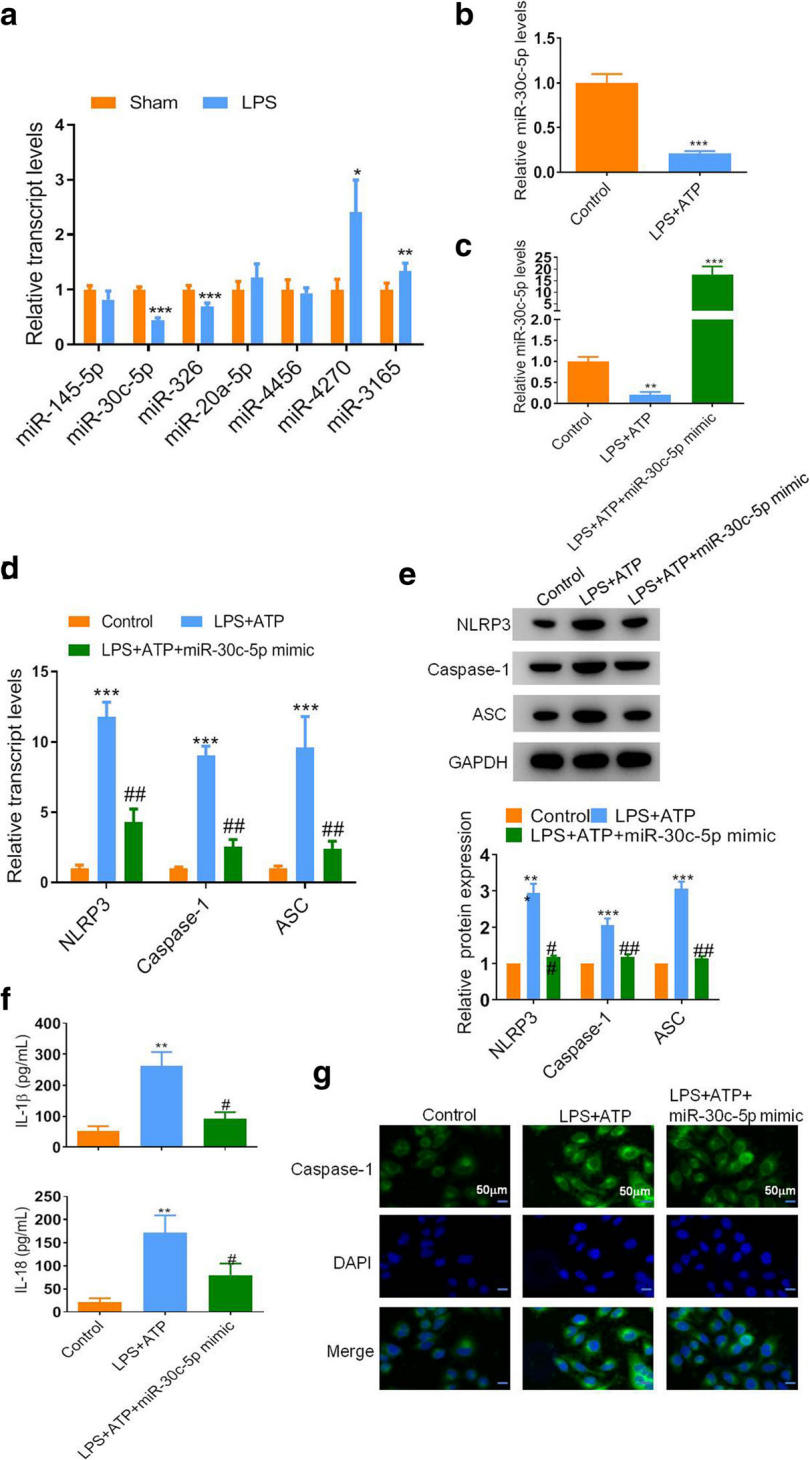
miR-30c-5p was elevated in the SAKI models and associated with the epithelial cell pyroptosis. **a** qRT-PCR analysis of the miRNAs expression in renal tissues of mice after LPS stimulation. **b** The expression level of miR-30c-5p in the cells after different treatments was detected by qRT-PCR. **c** The transfection efficiency of miR-30c-5p. **d** The relative mRNA expression levels of NLRP3, caspase-1, and ASC were performed by qRT-PCR. **e** Representative blots and statistical analysis were shown for the protein level of NLRP3, caspase-1, and ASC in HK-2 cells after different treatments. **f** ELISA results were presented for IL-1β and IL-18 expression in HK-2 cells. **g** Representative images of IF assay were shown for the expression of caspase-1 in HK-2 cells after different treatments. Nuclei were stained by DAPI. Bar, 50 μm. The data represented the mean ± SD of three biological replicates, *p < 0.05, **p < 0.01, and ***p < 0.001 *vs.* the control group or the sham group. #*p* < 0.05, ##*p* < 0.01, and ###*p* < 0.001 *vs.* the LPS + ATP group.

In order to study the role of miRNAs in sepsis-induced AKI, we measured the levels of seven miRNAs such as miR-145-5p, miR-30c-5p, miR-326, miR-20a-5p, miR-4456, miR-4270, and miR-3165 in the renal tissues, which were associated with the AKI formation. miR-30c-5p was one of the high-graded downregulated miRNAs, with almost 50% reduction in the septic mice than the sham ones (Fig. 2a). Moreover, we stimulated the septic condition in HK-2 cells using LPS + ATP treatment and found that the expression level of miR-30c-5p was also pronounced downregulated in the septic cells than the control group (Fig. 2b). Based on these findings, we hypothesized that miR-30c-5p was involved in the sepsis-induced AKI formation. For this purpose, we then regulated the miR-30c-5p expression in HE-2 cells through transfecting cells with miR-30c-5p mimic or related control (mimic-NC). The result showed that with the miR-30c-5p mimic transfection, the miR-30c-5p level was elevated almost 20 folds as compared with the control group (Fig. 2c). Besides, as shown in Fig. 2d, the mRNA levels of NLRP3, caspase-1, and ASC were increased in LPS + ATP-treated cells. These molecular alterations were further confirmed by western blot, which also showed a significant upregulation on the expression of NLRP3, caspase-1, and ASC in the LPS + ATP group than the control ones. Consistently, a higher IL-1β and IL-18 expression levels were observed in the supernatant of LPS + ATP-treated cells. Moreover, immunofluorescence analysis showed that caspase-1 expression was remarkably increased after LPS + ATP administration. However, the experiment where miR-30c-5p was overexpressed in the model cells showed a significant decrease in the level of these markers (Fig. 2 d, e, f, and g). These results indicated that miR-30c-5p might be a key mediator for sepsis-induced AKI regulation.

### TXNIP Was a Target of miR-30c-5p

**Fig. 3:**
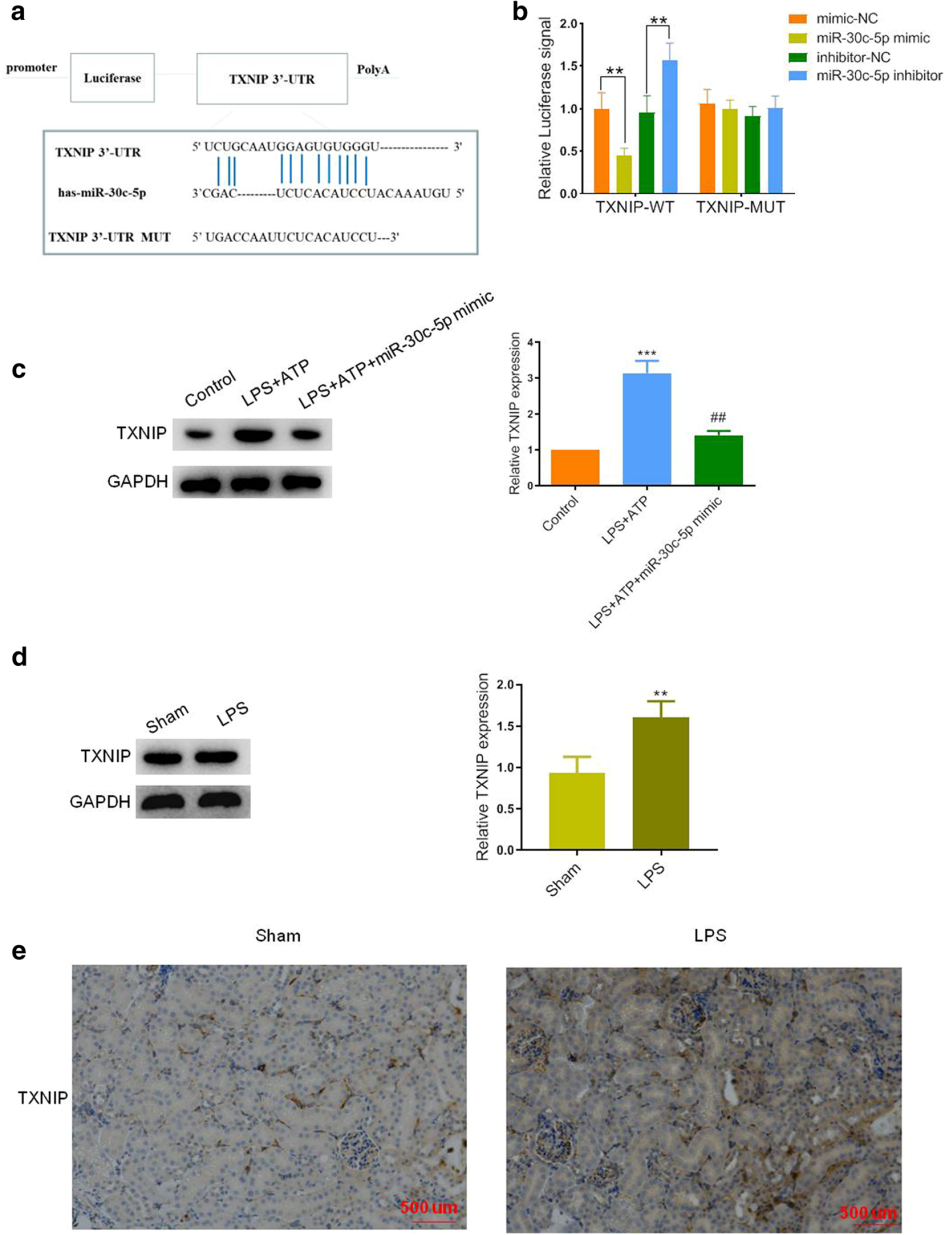
TXNIP was a target of miR-30c-5p. **a** The binding sites between miR-30c-5p and TXNIP were predicted using the TargetScan database. **b** The activity of luciferase reporters containing wild-type (WT) or mutant (MUT) TXNIP 3′-UTR were utilized with miR-30c-5p mimics, inhibitor, or respective controls to transfect the HEK-293 T cells. *p < 0.05, **p < 0.01, and ***p < 0.001. **c** Representative blots and statistical analysis were shown for the protein level of TXNIP in HK-2 cells after LPS + ATP, LPS + miR-30c-5p mimic, or control treatment. *p < 0.05, **p < 0.01, and ***p < 0.001 *vs.* the control group. #*p* < 0.05, ##*p* < 0.01, and ###*p* < 0.001 *vs.* the LPS + ATP group. **d** Representative blots were shown for the protein level of TXNIP in sham or LPS-treated mice. *p < 0.05, **p < 0.01, and ***p < 0.001 *vs.* the sham group. **e** Representative IHC image of TXNIP in the kidney tissues of LPS-induced sepsis mice. Bar, 500 μm. The data represented the mean ± SD of three biological replicates.

In general, the typical function of miRNAs was inhibited one or multiple target genes *via* binding to the 3’-UTR. Thus, in this study, TargetScan database was used to predict the potential targets of miR-30c-5p. TXNIP was one of the great-grade genes, and the binding sites between TXNIP and miR-30c-5p were shown in Fig. 3a. Besides, a luciferase report assay was performed to verify this relationship. As shown in Fig. 3b, miR-30c-5p mimic decreased the luciferase activity of wild-type TXNIP, while there was no significant change in mutant TXNIP. However, the miR-30c-5p inhibitor dramatically upregulated the wild-type TXNIP rather than the mutant one, indicating that TXNIP was a direct target gene of miR-30c-5p. Next, we further discussed the role of miR-30c-5p *in vitro*. As shown in Fig. 3c, the expression level of TXNIP was inclined almost 3 folds in the model group, and re-downregulated in the model + miR-30c-5p mimic group. We then next detected the TXNIP expression *in vivo*. The western blot analysis demonstrated that LPS treatment could incline the expression of TXNIP. A similar trend of this gene was observed in the kidney tissues of septic mice by IHC assay compared with the sham ones (Fig. 3 d and e). This data indicated that the expression of TXNIP was associated with the SAKI formation and regulated by miR-30c-5p.

### miR-30c-5p Inhibited Cell Pyroptosis *via* Regulating TXNIP Expression *In Vitro*

**Fig. 4:**
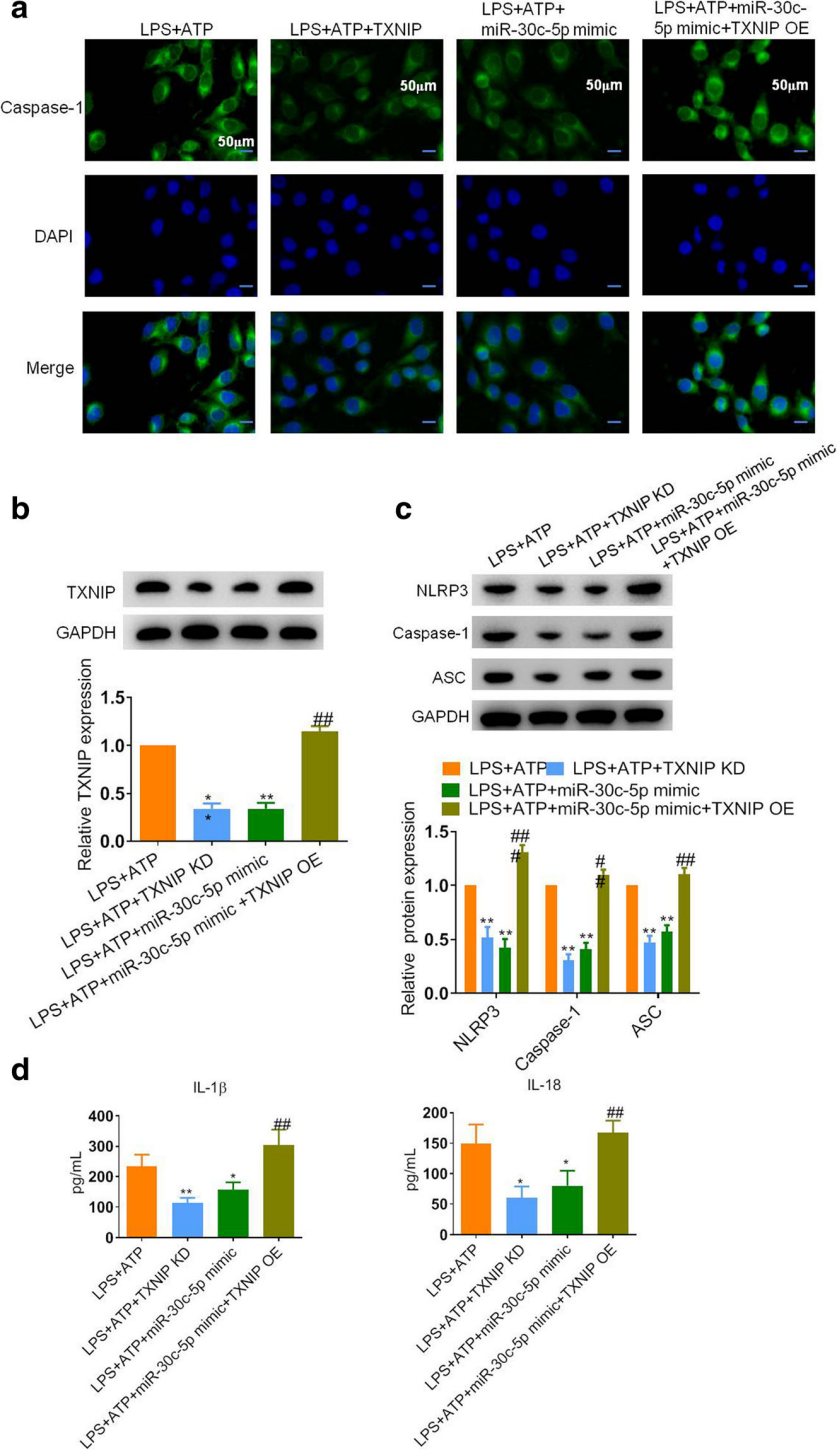
MiR-30c-5p inhibited LPS-induced pyroptosis *via* regulating TXNIP expression *in vitro*. **a** Representative images of IF assay were shown for the expression of caspase-1 in HK-2 cells after different treatments. Nuclei were stained by DAPI. Bar, 50 μm. **b** and **c** Representative blots and statistical analysis were shown for the protein level of TXNIP, NLRP3, caspase-1, ASC, and GAPDH in HK-2 cells after different treatments. **d** The content of IL-1β and IL-18 in cell supernatant was detected by ELISA. The data represented the mean ± SD of three biological replicates. *p < 0.05, **p < 0.01, and ***p < 0.001 *vs.* the LPS + ATP group. #*p* < 0.05, ##*p* < 0.01, and ###*p* < 0.001 *vs.* the LPS + ATP + miR-30c-5p mimic group.

Next, we sought to explore the role of the miR-30c-5p/TXNIP axis in HK-2 cell pyroptosis upon LPS + ATP stimulation. As shown in Fig. 4a, LPS + ATP treatment promoted the expression of caspase-1 inside of the HK-2 cells, the silence of TXNIP or overexpression of miR-30c-5p both decreased caspase-1 levels, while when we transfected the cells with miR-30c-5p mimic and TXNIP-OE vector before LPS + ATP administration, the expression level of caspase-1 was re-inclined. At the same time, a similar trend was determined in the protein levels of TXNIP, NLRP3, caspase-1, and ASC (Fig. 4 b and c). Consistently, the downstream cytokines, including IL-1β and IL-18, were both decreased no matter with TXNIP-KD or miR-30c-5p mimic transfection compared with the LPS + ATP group. However, the experiments where TXNIP and miR-30c-5p were both overexpressed showed a significant increase in the level of these molecules (Fig. 4d).

### miR-30c-5p Inhibited Cell Pyroptosis *via* Regulating TXNIP Expression *In Vivo*

**Fig. 5:**
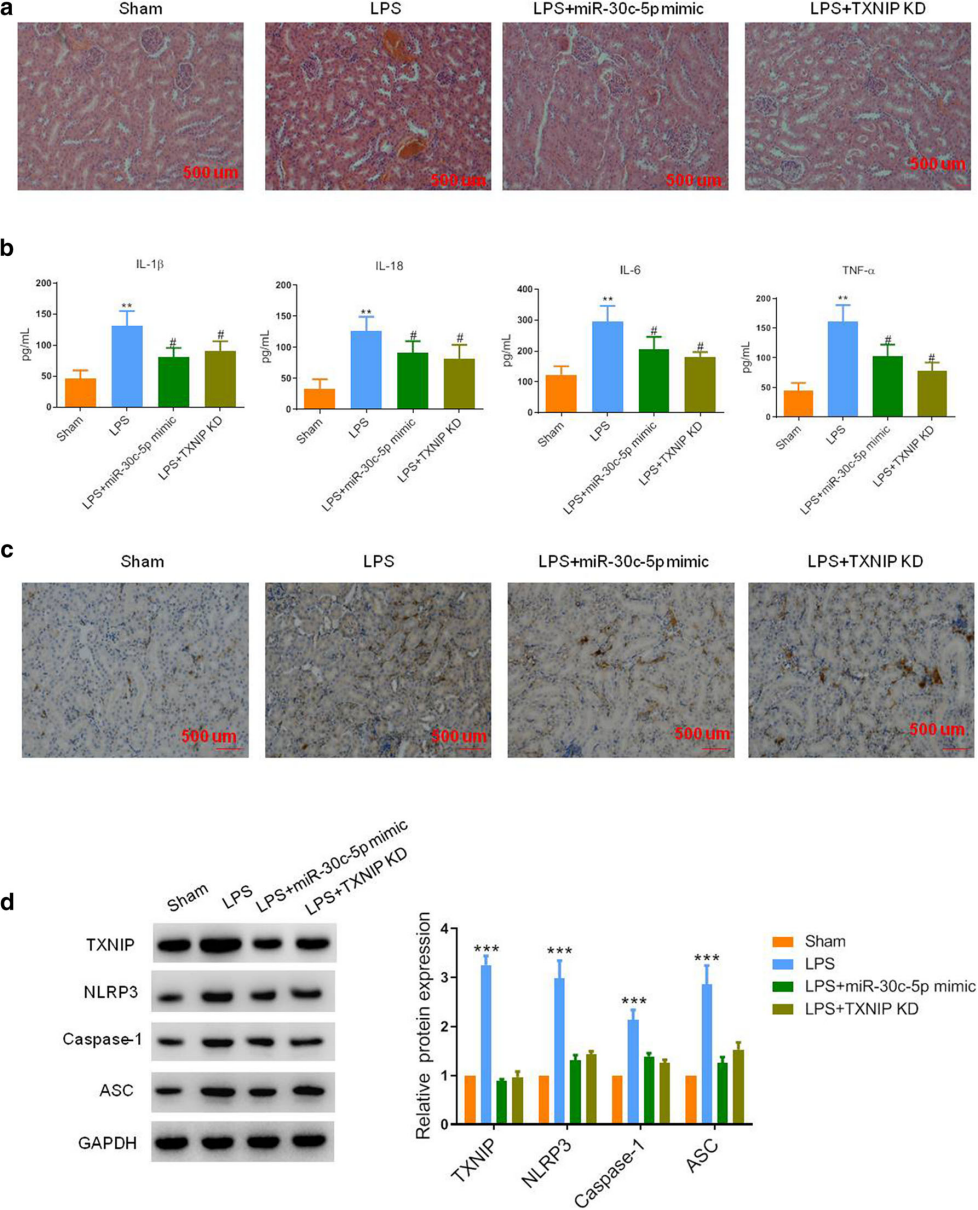
MiR-30c-5p inhibited LPS-induced pyroptosis *via* regulating TXNIP expression *in vivo*. **a** Representative H&E images were shown for the kidney tissue after different treatments. Bar, 500 μm. **b** The concentration of serum IL-18, IL-6, IL-1β, and TNF-α in mice after different treatments were determined by ELISA assay. **c** Representative images of IHC assay of TXNIP in the kidney tissue after different treatments. Bar, 500 μm. **d** Representative blots and statistical analyses were shown for the protein level of TXNIP, NLRP3, caspase-1, ASC, and GAPDH in the renal tissue of mice after different treatments. The data represented the mean ± SD of three biological replicates, *p < 0.05, **p < 0.01, and ***p < 0.001 *vs.* the sham group. #*p* < 0.05, ##*p* < 0.01, and ###*p* < 0.001 *vs.* the LPS group.

To determine whether increased miR-30c-5p under LPS stimulation would result in a protective effect against AKI, kidney-specific overexpression of miR-30c-5p was performed. The mice that had undergone kidney-specific injection of miR-30c-5p mimic demonstrated less tubular cavity expansion, and vacuolar degeneration of tubular epithelial cells and the expression level of inflammatory factors were dramatically declined as compared with the control mice (Fig. 5 a and b). Compared with the LPS group, overexpression of miR-30c-5p markedly inhibited the expression of pyroptosis markers, including TXNIP, NLRP3, caspase-1, and ASC in the renal tissues, suggesting that miR-30c-5p suppressed cell pyroptosis in the kidney during AKI development (Fig. 5 c and d). Furthermore, to validate whether TXNIP was responsible for the kidney injury, kidney-specific inhibition of TXNIP was also performed. We found that the silence of TXNIP in the kidney exerted a notable protective effect against kidney injury. Besides, the downregulation of TXNIP also significantly downregulated the expression of pyroptosis-related genes, suggesting that TXNIP played an essential role in the initiation of pyroptosis during AKI formation (Fig. 5).

## DISCUSSION

Sepsis has been considered as causative for SAKI. During the development of sepsis, several inflammatory cytokines, including IL-1, TNF-α, and IL-6, are released and thus initiate downstream pathways that lead to cell death and organ disorder [19]. Currently, researchers reported that the injury and death of renal tubular cells including apoptosis, necroptosis, and autophagy are the critical events in the progression of SAKI. Therefore, elucidating the underlying molecular basis of tubular cell death is still required [20]. As a novel form of inflammatory-related cell death, more interests have a focus on pyroptosis in kidney cells. For instance, one group found the specific characteristics of pyroptosis in tubular epithelial cells. They revealed that LPS treatment increased the expression of pyroptosis-related marker caspase-11 and GSDMD *in vivo* and *in vitro*, and hence aggravated the damage of the kidney [21]. Besides, in the study of Lu *et al.*, a high level of cleaved GSDMD, accompanied by increased IL-18 excretion, was detected in tubular cells, which is responsible for the renal function dysregulation in AKI [22]. Consistently, in this study, we also found the pyroptosis occurred in renal tissues during SAKI, as evidenced by an upregulated cleaved GSDMD expression, activated NLRP3/caspase-1 pathway-related genes, and increased downstream inflammatory cytokines such as IL-18 and IL-1β, in the mice subjected to LPS compared with the sham group. Moreover, when treated the HK-2 cells with LPS + ATP *in vitro*, NLRP3/caspase-1-dependent pyroptosis was also observed. These findings indicated that HK-2 cell pyroptosis might be a critical event during SAKI progression.

Previous studies have demonstrated that miRNAs are dysregulated in septic patients, are contributed to the survival rate, and presented a key function for septic disease prognosis [23]. Based on these findings, we sought to discover the specific miRNA which is associated with the formation of SAKI in this study. The miR-30c-5p is dramatically downregulated in SAKI patients, but the mechanisms remain unknown [17]. Our work here identified that miR-30c-5p was decreased in the LPS-induced septic models. Moreover, transfection of miR-30c-5p mimic had the potential ability to suppress on the level of TNF-α and IL-6 *in vivo* and presented a protecting effect on the kidney against sepsis-induced injury, indicating that miR-30c-5p could be a novel target for SAKI therapy. In addition, overexpression of miR-30c-5p inhibited NLRP3 activation, thereby suppressing the secretion of IL-18 and IL-1β, as well as inhibiting ASC and caspase-1 expression both *in vivo* and *in vitro*. Further, through the bioinformatic analysis and luciferase assay, we revealed that miR-30c-5p controlled the HK-2 cell pyroptosis *via* directly targeting TXNIP.

It has been reported that TXNIP is an important mediator for NLRP3 inflammasome activation, emerged as a target in multiple diseases, including cervical inflammation, diabetic nephropathy, and tubular injury [24–26]. Inhibition of TXNIP declines the abundance of NLPR3 and caspase-1, thereby reduces IL-18 and IL-1β excretion [27]. In this study, we found miR-30c-5p suppressed the TXNIP, which promoted NLRP3 inflammasome formation and stimulation caspase-1 cleavage. While when we further overexpression of TXNIP reversed the effect of miR-30c-5p, which activated NLRP3-mediated pyroptosis. Moreover, in our *in vivo* study, the silence of TXNIP markedly decreased the expression level of NLRP3/caspase-1 pathway-related genes and alleviated the damage of the kidney under the sepsis conditions, which is similar with the result of miR-30c-5p treatment. Collectively, TXNIP-mediated NLRP3 inflammasome cascade response is considered as a crucial signal pathway in the involvement of miR-30c-5p in the process of SAKI.

In summary, our study provided evidence that NLRP3 inflammasome-related pyroptosis occurred in SAKI. Moreover, miR-30c-5p served as a key regulator of NLRP3 pathway-associated pyroptosis in SAKI *via* directly binding to TXNIP. Our findings revealed that miR-30c-5p might act as a new biomarker and therapeutic target for patients with SAKI.
